# Amyand’s Hernia With Retractile Testis and a Normal Appendix: Is Appendectomy Truly Required?

**DOI:** 10.7759/cureus.84303

**Published:** 2025-05-17

**Authors:** Susheel Sathvik Palasanadram Ravikumar, Nithya Shekar, Kalpana Vineet, Sagar N Patil, Aiswarya Ravi

**Affiliations:** 1 General Surgery, Vydehi Institute of Medical Sciences and Research Centre, Bengaluru, IND; 2 Department of Surgery, Kalinga Institute of Medical Sciences, Bhubaneswar, IND

**Keywords:** amyand’s hernia, appendicitis, case report, inguinal hernia, pediatric surgery, retractile testis

## Abstract

An inguinal hernia is a common surgical condition in which abdominal contents protrude through the lower abdominal wall into the inguinal canal. The hernia sac can contain various structures, including the appendix, which, when present, is referred to as Amyand's hernia. The condition is rare, with a normal appendix found in less than 1% of inguinal hernias. This condition was first described by Claudius Amyand in 1735. The coexistence of the appendix and a testis (either undescended or retractile) within the hernia sac is a rare phenomenon, adding to the complexity of diagnosis and management. In this report, we have a one-year-old male infant presenting with a right-sided irreducible inguinal hernia associated with swelling and pain for four hours. Physical examination and ultrasound suggested an irreducible hernia with omentum as the content, but without evidence of appendix or testis involvement. On surgical exploration, a normal appendix and a retractile right testis were found within the hernial sac. The hernia contents were reduced, and a right inguinal herniotomy was performed. Intraoperative and postoperative periods were uneventful. The patient was discharged after three days and had an unremarkable follow-up. Amyand's hernia is an uncommon condition in which the appendix is located within the inguinal hernia sac. The presence of both the appendix and a testis in the same sac is even rarer. Our case differs from the classic presentation of Amyand's triad, as it involves a retractile testis and a normal appendix. This highlights the need for thorough clinical evaluation and tailored treatment based on individual findings. Surgeons should be aware of the varied presentations of this condition and the recommended management strategies to provide optimal care.

## Introduction

An inguinal hernia is defined as the protrusion of abdominal contents through the lower abdominal wall into the inguinal canal [1,2]. Despite being the most commonly performed surgical procedure, inguinal hernias often present with atypical features, especially regarding the contents of the hernia sac. Inguinal hernias can contain a wide variety of structures, such as segments of bowel, omentum, ovaries, bladder, Meckel’s diverticulum, and, occasionally, the appendix. The presence of the vermiform appendix, regardless of whether it is inflamed, within the hernia sac is referred to as Amyand's hernia [3-6]. The presence of a normal appendix within an inguinal hernia sac is an exceptionally rare occurrence, reported in approximately 0.5% to 1% of cases [2]. This condition is named after Claudius Amyand, who first described it in 1735 in an 11-year-old male patient with a discharging fecal fistula that traced to a perforated appendix located within the hernia sac. Here, we report a case of a one-year-old male baby presenting with right-sided irreducible congenital inguinal hernia, which on exploration was found to have right retractile testis with normal appendix as its contents.

## Case presentation

A one-year-old male baby presented to the emergency department with painful inguinal swelling in the right groin for four hours. The patient had no h/o vomiting/fever in the past week. Abdominal pain was colicky in type, severe in intensity, and non-radiating with no identifiable aggravating or relieving factors. The patient had a history of a similar, non-tender, reducible swelling for the past six months, which intermittently appeared during activities such as playing or crying. Previously, the swelling would spontaneously reduce with rest or sleep; however, in the past four hours, it remained persistent and irreducible. The patient did not have any history of jaundice, urinary, or bowel complaints.

**Figure 1:**
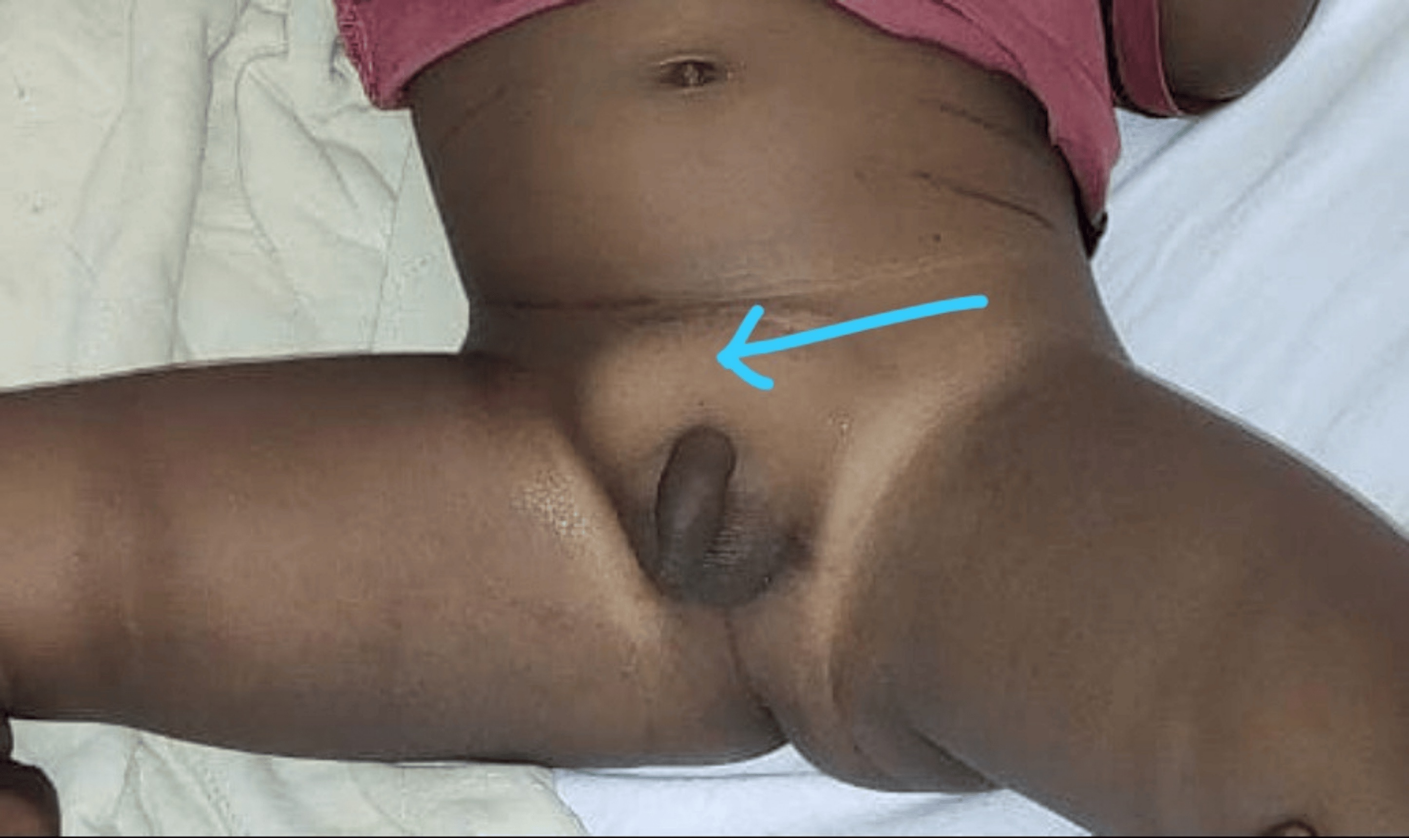
Clinical photo at the time of presentation The arrow indicates the site of swelling.

The abdomen was soft, non-distended on physical examination. A swelling measuring 3 x 2 cm in the right inguinal region, extending from the deep inguinal ring to the root of the scrotum, was tender and non-reducible, as shown in Figure 1. The right testis was palpable near the root of the scrotum, comes down on pulling, and goes back in its own (retractile testis). The chair test could not be performed. Clinically, the baby was diagnosed to be a case of right-sided irreducible congenital inguinal hernia.

Investigations

**Figure 2:**
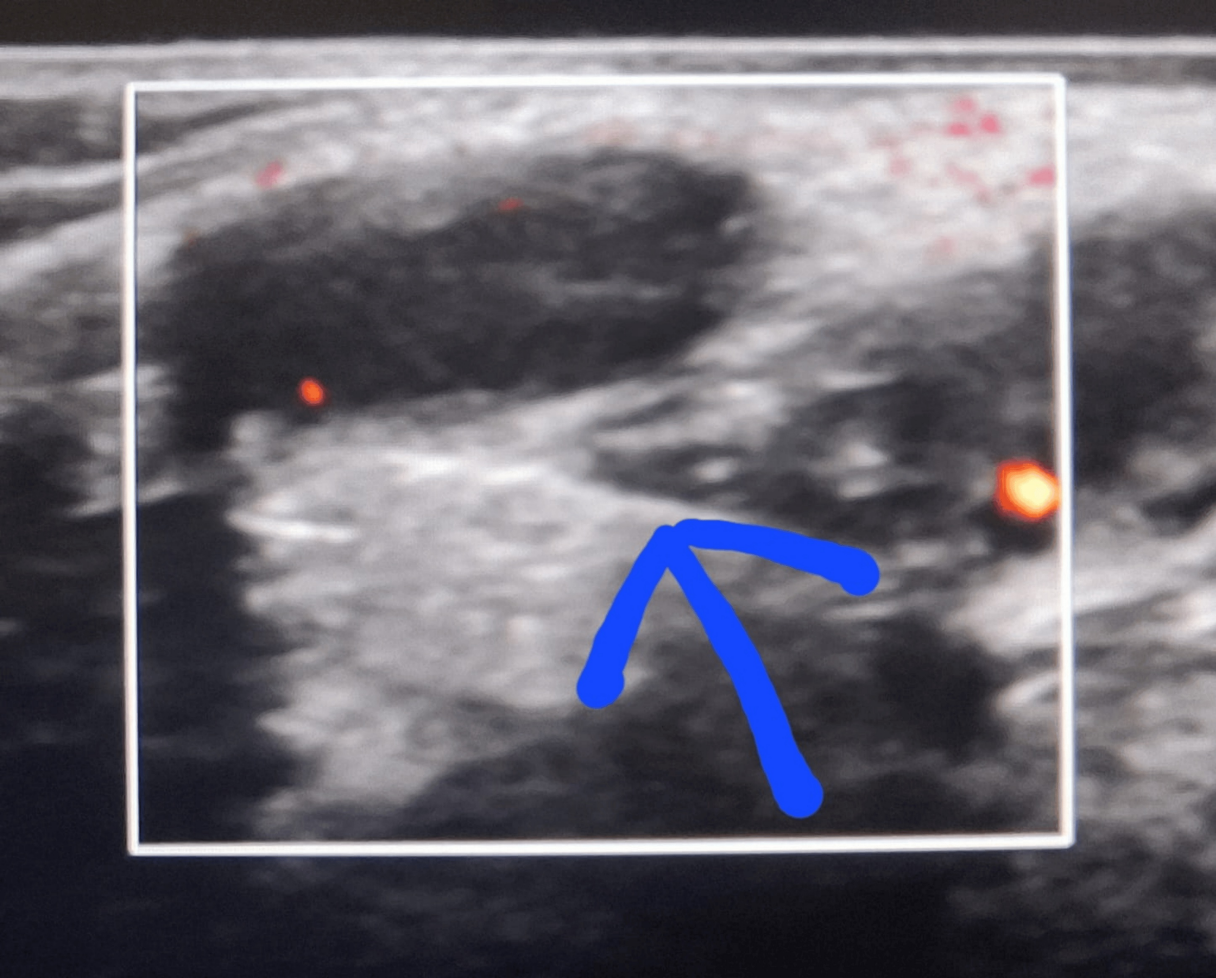
Ultrasound of the right inguinoscrotal region The arrow shows the defect in the deep inguinal ring.

Laboratory investigations were normal. Inguinoscrotal ultrasound with Doppler showed an inguinal hernia with omentum as content and no evidence of bowel loops, appendix, or testis as contents, as shown in Figure 2.

**Figure 3:**
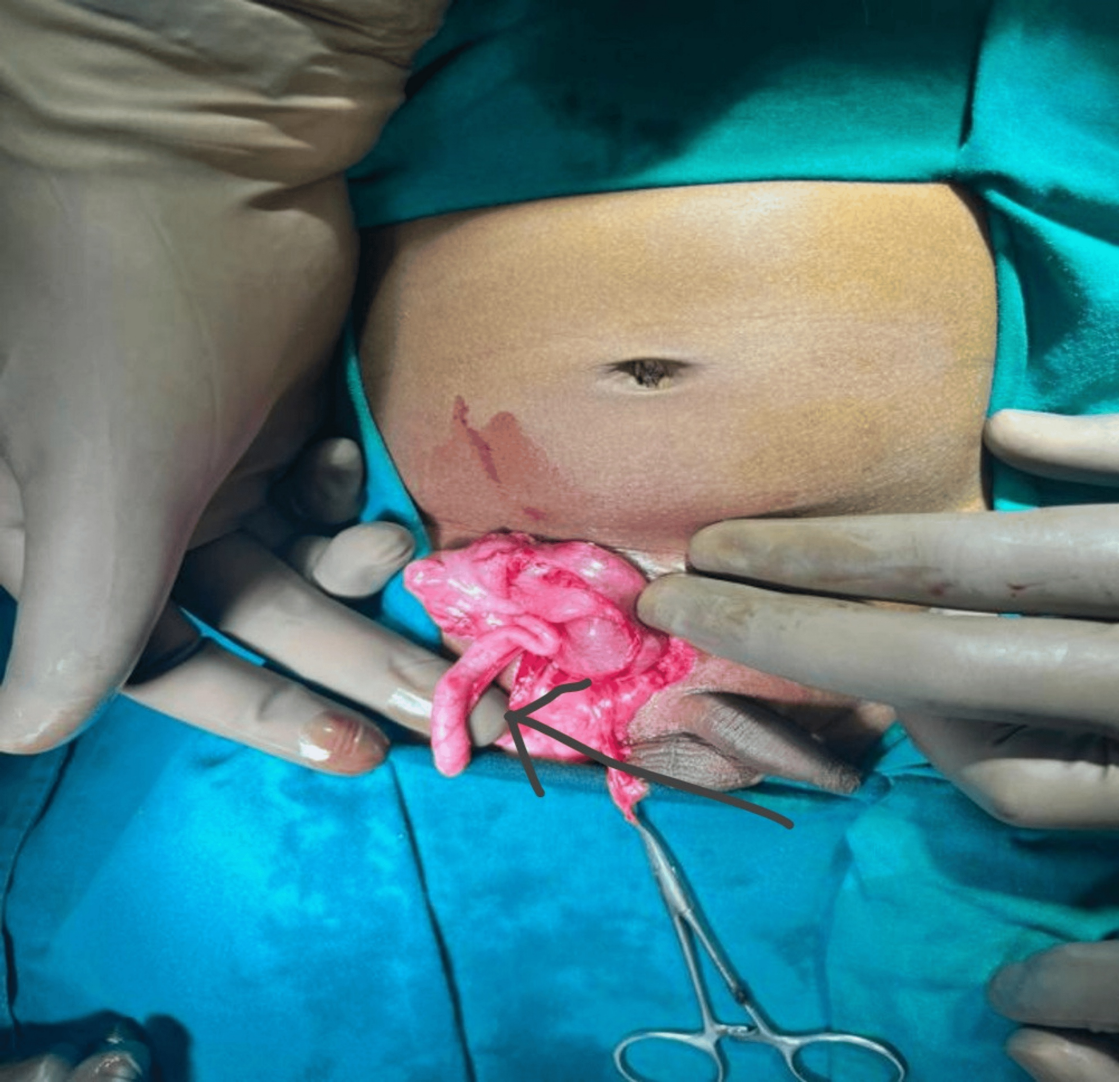
Intraoperative photo showing the appendix (shown by the arrow)

**Figure 4:**
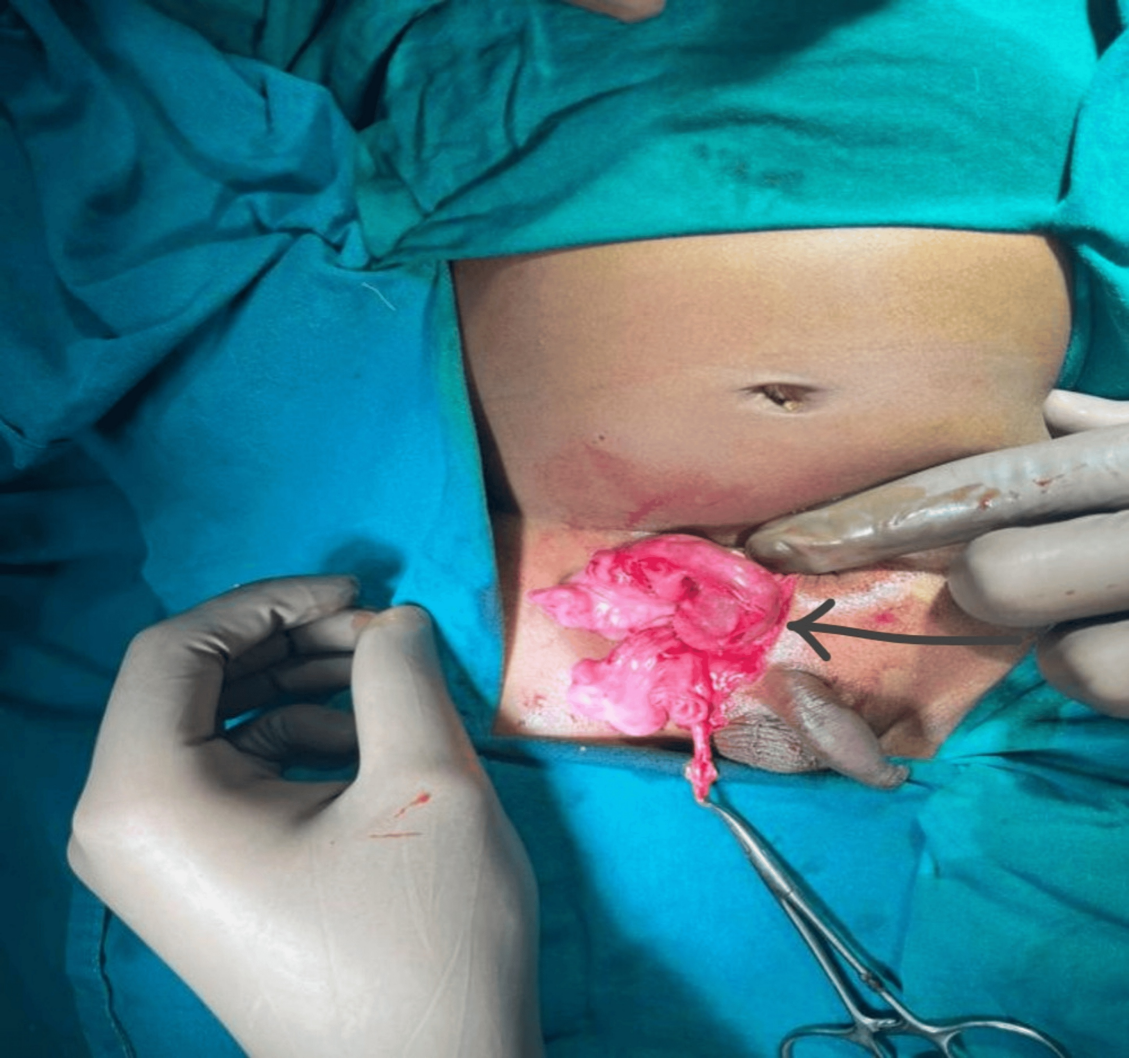
Intraoperative photo showing the testis The arrow shows the right testes in the right inguinal region.

After proper consent, the patient was then taken up for exploration of the inguinoscrotal region. Intraoperative findings included the ileocecal junction with an appendix without inflammation (Figure 3) and right-sided testis noted in the inguinal canal (Figure 4).

Both the appendix and the testis were normal in appearance. The contents were reduced, and right inguinal herniotomy was performed. Intraoperative and postoperative periods were uneventful. The patient was discharged after three days and had an unremarkable follow-up.

## Discussion

Inguinal hernias may contain a diverse range of anatomical structures, including segments of the bowel, omentum, ovaries, urinary bladder, Meckel’s diverticulum, testes, and, on rare occasions, the appendix [5,6]. Amyand’s hernia was first described in 1735 in an 11-year-old male patient with a discharging fecal fistula that traced to a perforated appendix located within the hernia sac, and an appendectomy was performed successfully [7]. This condition is regarded as relatively uncommon, with an estimated prevalence of about 1% among incarcerated inguinal hernias. Often, it is diagnosed incidentally during surgical repair of the hernia [3].

The term Amyand’s hernia refers to a spectrum of clinical presentations involving the presence of the appendix within an inguinal hernia sac. These scenarios include (a) an inflamed appendix within the hernia sac, which may present with symptoms resembling acute appendicitis or complicate the surgical management of the hernia; (b) a perforated appendix within the sac, potentially resulting in severe complications such as peritonitis or abscess formation, and requiring prompt surgical intervention; and (c) a non-inflamed appendix contained within an irreducible inguinal hernia, which may remain asymptomatic and is often identified incidentally during hernia repair surgery, as in our case, or through imaging performed for unrelated reasons [2,8].

The incidence of appendicitis occurring within an inguinal hernia sac is exceptionally rare, estimated at 0.07-0.13%. Similarly, the occurrence of a perforated appendix within an incarcerated inguinal hernia is also infrequent, accounting for approximately 0.1% of all cases of appendicitis [9]. Similarly, the occurrence of appendix and retractile testis within an incarcerated inguinal hernia is also infrequent. The majority of Amyand's hernia cases are seen in men, particularly those with right-sided inguinal hernias, with groin pain being the most common presenting symptom [3]. Amyand's hernia does not show a specific age predilection, as it has been reported in individuals ranging from neonates to those as old as 92 years [9].

Definitive preoperative diagnosis of Amyand’s hernia is uncommon, as it is most frequently identified incidentally during surgical exploration for inguinal hernia repair [3,9]. Computed tomography (CT) is regarded as the most effective imaging modality for the evaluation of acute abdominal conditions, including the assessment of abdominal wall hernias [6]. Inguinal hernias are traditionally diagnosed based on clinical examination or intraoperative findings; thus, imaging modalities may have limited utility in the differential diagnosis, particularly in straightforward cases [9]. In fact, establishing a preoperative clinical diagnosis of Amyand’s hernia has been reported as virtually impossible [10]. However, imaging modalities such as ultrasonography and computed tomography (CT) may offer some diagnostic assistance [11,12]. Notably, previous studies on diagnostic approaches have often lacked comprehensive data regarding sensitivity and specificity [10].

In the management of Amyand’s hernia, the surgeon must address both the hernia itself and, if present, the appendicitis. In cases where the appendix is non-inflamed, a critical consideration is whether a prophylactic appendectomy is warranted, as well as the appropriateness of using prosthetic mesh for hernia repair [13]. Several studies have proposed that prophylactic appendectomy may not be needed if the appendix appears normal and does not exhibit signs of inflammation [3]. However, Ofili’s report highlighted two cases in which acute appendicitis developed following inguinal hernia repair performed without an incidental appendectomy. By contrast, it was also noted in 11 cases where no complications, such as wound infections or hernia recurrence, were observed following hernia repair combined with an incidental appendectomy [14].

Furthermore, it has been suggested that manipulation of the appendix during hernia repair, without its removal, might increase the risk of developing appendicitis [15]. This has led to recommendations for performing an incidental appendectomy in such cases [14]. However, the decision to remove a normal appendix remains a clinical dilemma, as there is currently no evidence-based consensus or definitive guidelines to inform this decision [1,2].

Lonsanoff et al. introduced a classification system in 2008 for Amyand's hernias, which proves useful for guiding intraoperative decision-making [16] (Table 1). The modified Losanoff-Basson classification of Amyand’s hernia and its management in children was proposed for guiding surgeons in 2023 for making informed decisions for the management of Amyand’s hernia in children [17] are presented in Table 2.

**Table 1:** Losanoff-Basson classification of Amyand’s hernia (AH) and their management in adults (2008). Permission was taken from the original article to publish the table [16].

Classification	Description	Surgical management
Type I	Normal appendix in inguinal hernia	Hernia reduction, mesh repair, and appendectomy in young patients
Type II	Acute appendicitis within an inguinal hernia and no abdominal sepsis	Appendectomy through hernia, primary repair of hernia, no mesh
Type III	Acute appendicitis within an inguinal hernia or the abdominal wall or peritoneal sepsis	Laparotomy, appendectomy, primary repair of hernia, no mesh
Type IV	Acute appendicitis within an inguinal hernia with related or unrelated abdominal pathology	Management as detailed above for hernia types I–III and treat the second pathology as appropriate

**Table 2:** Modified Losanoff-Basson classification of Amyand’s hernia (AH) and their management in children. Permission was taken from the original article to publish the table [17].

Features	Surgical management
Normal appendix within the right inguinal hernial sac	Reduction of the appendix with open/laparoscopic hernial repair
Acute appendicitis in an inguinal hernial sac with no abdominal sepsis and/or dense adhesions of the appendix with a hernial sac or left-sided AH with or without acute appendicitis	Appendectomy and hernial repair through an inguinal approach/
	Laparoscopic appendectomy and hernial repair
Complicated acute appendicitis in an inguinal hernial sac with abdominal sepsis	Appendectomy through laparotomy/laparoscopy with hernial repair
AH with acute appendicitis associated with concomitant abdominal pathology	Laparotomy/laparoscopy with appendectomy, and management of concomitant disease
Recurrent inguinal hernia with adhesion of the appendix to the hernial sac	Appendectomy, herniotomy with narrowing of the deep ring, and post-wall repair if needed

It has been observed that appendices located within inguinal hernia sacs are more prone to inflammation compared to those in the abdominal cavity [3]. One potential explanation for this increased incidence of inflammation is that the appendix, when positioned within the hernia sac in the inguinal canal, is more susceptible to injury and secondary inflammatory processes [3,9,12]. Moreover, intermittent compression of the appendix by contracting abdominal musculature may compromise its vascular supply, potentially resulting in ischemia, infection, and subsequent severe inflammation [3]. Kose et al. reported a series of five cases of Amyand’s hernia in which hernia repair with mesh placement was performed, accompanied by the removal of non-inflamed appendices. The authors proposed that fibrous adhesions between the appendix and the hernia sac, combined with surgical dissection and manipulation of the appendix, may contribute to inflammation and potentially precipitate secondary appendicitis [18].

However, it is important to note that the mere presence of the appendix within the hernia sac does not invariably lead to the development of appendicitis [3]. Preoperative assessment of appendiceal adherence to the hernia sac is not feasible with current diagnostic modalities. Consequently, the surgical approach is often guided by the surgeon’s experience and intraoperative findings. Decisions regarding the performance of a prophylactic appendectomy should be based on sound clinical judgment, with careful consideration of the potential risks and benefits associated with the procedure [2].

Elective repair of inguinal hernias using a tension-free technique with prosthetic mesh is widely considered the gold standard, as it significantly reduces the risk of recurrence when compared to traditional repairs utilizing native tissue reinforcement [9]. Although inguinal hernia repair is generally classified as a clean surgical procedure, the addition of a prophylactic appendectomy reclassifies it as a clean-contaminated operation [13]. In such cases, the use of prosthetic mesh is generally avoided to minimize the risk of postoperative infection. Nevertheless, the appropriateness of mesh placement in the context of incarcerated hernias remains a subject of ongoing debate within the surgical community.

Lie et al. reported that the occurrence of surgical site infections did not invariably result in mesh-related infections. They concluded that, provided the surgical field remains clean and free of contamination, the use of prosthetic mesh is not necessarily contraindicated [19]. In our patient's case, although the surgery was performed as an emergency procedure, the contents were reduced easily after careful dissection. The fibrous attachment between the appendix and the hernia sac was successfully separated without any intraoperative complications. Based on these measures and the patient being a one-year-old baby, a herniotomy would suffice, and the appendix was normal and hence saved.

The most frequently utilized surgical approach for the management of Amyand's hernia involves performing an appendectomy via herniotomy [9]. In recent years, laparoscopic surgery has become increasingly utilized for these cases [9]. Vermillion et al. reported the inaugural case of a laparoscopic appendectomy performed in the context of an Amyand's hernia complicated by appendicitis [20]. Laparoscopy provides both diagnostic and therapeutic benefits, enabling the assessment of the hernia type, the condition of the appendix, and the overall status of the hernia [21]. However, there is no established consensus on the most effective management approach [21].

When the testis is found alongside the appendix within the hernia sac, the decision to proceed with orchidopexy or orchidectomy is determined by the viability of the testis [22]. In our case, the testis was retractile, and as such, we opted to release the adhesions, allowing the testis to return to the base of the scrotum. Consequently, no orchidopexy or orchidectomy was required.

The presence of both a testis and an appendix within a hernia sac is a rare occurrence, with only a limited number of cases reported in the literature [23]. When Amyand’s hernia, an undescended testis, and appendicitis are found together, this combination can be referred to as Amyand’s triad [23]. However, our case does not fit the typical definition of Amyand’s triad, as it involves a retractile testis and a normal appendix. This highlights the necessity for a careful clinical assessment, radiological and laboratory investigations, and appropriate, tailored treatment based on the individual findings.

The primary complications associated with Amyand's hernia include appendix perforation, necrotizing fasciitis of the abdominal wall, and secondary intestinal perforation [3]. Amyand’s hernias are associated with a high mortality rate, ranging from 14% to 30%, and are closely linked to the peritoneal dissemination of sepsis. In the present case, emergency surgery was deemed necessary for the management of the incarcerated Amyand's hernia, as manual reduction was not feasible and to avert the risk of further complications [24].

## Conclusions

The simultaneous presence of both the appendix and a testis (whether undescended or retractile) within an irreducible inguinal hernia is a very rare condition with very few documented cases. When Amyand's hernia, an undescended testis, and appendicitis are found together, it can be referred to as Amyand's triad. However, our case does not align with the typical presentation of Amyand's triad, as it involves a retractile testis and a normal appendix. This highlights the importance of performing a comprehensive clinical assessment and offering personalized treatment based on the unique circumstances. Surgeons should be aware of this condition and its recommended management protocols to ensure the provision of appropriate and effective treatment.
